# Combination therapy of ipilimumab and nivolumab induced thyroid storm in a patient with Hashimoto’s disease and diabetes mellitus: a case report

**DOI:** 10.1186/s13256-018-1708-x

**Published:** 2018-06-19

**Authors:** Kazuko Yonezaki, Toshihiro Kobayashi, Hitomi Imachi, Takuo Yoshimoto, Fumi Kikuchi, Kensaku Fukunaga, Seisuke Sato, Tomohiro Ibata, Nao Yamaji, Jingya Lyu, Tao Dong, Koji Murao

**Affiliations:** Department of Endocrinology and Metabolism, Faculty of Medicine, Kagawa University, 1750-1, Miki-cho, Kita-gun, Kagawa 761-0793 Japan

**Keywords:** Thyroid storm, Immune checkpoint inhibitor, Hashimoto’s disease

## Abstract

**Background:**

Recently, immune checkpoint inhibitors have widely been used for the management of advanced melanoma. However, high-grade immune-related adverse events can occur, particularly with combination immunotherapy. We report a case of a patient with melanoma who developed thyroid storm following treatment with ipilimumab and nivolumab.

**Case presentation:**

An 85-year-old Japanese man with a history of malignant melanoma presented to our department with severe thyrotoxicosis and poor blood glucose control. He was already being treated for Hashimoto’s disease and type 2 diabetes mellitus before the treatment for the melanoma. During admission, laboratory investigations revealed the following thyroid functions: thyroid-stimulating hormone below sensitivity, free triiodothyronine 31.7 pg/ml, and thyroglobulin 48,000 IU/ml. Thyroid-stimulating hormone receptor antibody was negative, and a ^99m^Tc-labeled thyroid scan revealed a markedly decreased uptake. He was treated with beta-blocker, orally administered potassium iodine, a relatively low dose of prednisolone, and insulin injection therapy to control his blood glucose, resulting in an improvement in thyroid function and his symptoms.

**Conclusion:**

It might be important to be aware of the possibility of thyroid storm induced by immune checkpoint inhibitors.

## Background

Thyroid storm is an acute, life-threatening state induced by excessive release of thyroid hormones in individuals with thyrotoxicosis. The clinical symptoms include fever, tachycardia, and neurological and gastrointestinal (GI) abnormalities. Diagnosis is dependent on clinical symptoms, and no specific laboratory tests are available. Many factors including some drugs may precipitate the progression of thyrotoxicosis to thyroid storm [1]. In thyroid storm, a patient’s heart rate, blood pressure, and body temperature can get to dangerously high levels. Without prompt treatment, thyroid storm is often fatal.

Immune checkpoint inhibitors are widely used for the management of advanced melanoma. These include ipilimumab to block the negative checkpoint regulator cytotoxic T-lymphocyte antigen-4 (CTLA-4) on the surface of activated T cells [2]. Other immune checkpoint inhibitors were developed, including pembrolizumab and nivolumab; both of these immune checkpoint inhibitors are immunoglobulin G4 (IgG4) monoclonal antibodies that target the programmed cell death protein 1 (PD-1), which is a negative regulatory T cell surface receptor [3]. During the development of these immune checkpoint inhibitors, it was quickly recognized that the immune checkpoint inhibitors were associated with a novel syndrome of autoimmune or autoinflammatory side effects [4]. These toxicities have become known as immune-related adverse events (irAEs) and occur more frequently when these agents are administered in combination regimens [5]. In this report, we present a very rare case of thyroid storm in a patient with advanced melanoma receiving dual ipilimumab and nivolumab therapy and discuss the clinical presentation and therapeutic interventions in a patient with Hashimoto’s disease and type 2 diabetes mellitus.

## Case presentation

An 85-year-old Japanese man with a history of malignant melanoma of the nasal cavity presented to our department with severe thyrotoxicosis and poor blood glucose control. He had been treated for hypothyroidism secondary to Hashimoto’s disease and type 2 diabetes mellitus with insulin self-injection therapy before undergoing treatment of malignant melanoma. A surgical operation of his nasal cavity was done at first, followed by one of four planned cycles of nivolumab therapy. Subsequently, two courses of ipilimumab were given as standard therapy after nivolumab therapy. Two weeks later after receiving a dual course of ipilimumab as a third treatment, he presented with a fatigue, nausea, and sweating, which progressed to clinical and biochemical thyrotoxicosis. On admission to our hospital, he was febrile with a temperature of 38.0 °C, tachycardic, agitated, and acutely anxious but still conscious (restlessness). His Glasgow Coma Scale score was 14/15. His blood pressure had decreased to 70/50 mmHg.

His physical examination revealed a diffuse goiter without exophthalmoses. His abdomen was soft and non-tender and his skin was warm and wet. He was also diaphoretic with jugular venous distension and peripheral edema, and his chest was clinically clear. His medical history included hypothyroidism due to Hashimoto’s disease, diagnosed at 62 years of age and treated with thyroid hormone replacement, as well as type 2 diabetes mellitus treated by self-injection of insulin with a good glycemic control. He had both family histories of thyroid diseases and diabetes. An electrocardiogram showed marked tachycardia with atrial fibrillation, but a chest radiograph was normal.

The laboratory data are shown in Table 1. Laboratory investigation revealed the following thyroid function results: thyroid-stimulating hormone (TSH) below sensitivity, free triiodothyronine (FT3) 31.7 pg/ml, and free thyroxine (FT4) 3.43 ng/dl. Remarkably, his thyroglobulin was elevated to 48,000 IU/ml. TSH receptor antibody was negative, and a ^99m^Tc-labeled thyroid scan revealed a markedly decreased uptake (Tc retention index-uptake ratio 0.0%; normal range 0.4–3.0). His blood glucose was markedly elevated; in which case, we needed to increase the amount of insulin to control his blood glucose. Further immunological investigation revealed normal serum levels of anti-glutamic acid decarboxylase (GAD) antibody, anti-insulinoma antigen 2 (IA-2) antibody, and insulin autoantibody (IAA).

**Table 1:** A summary of laboratory data

Blood chemistry		Reference
C-reactive protein (mg/dl)	11.6	≤ 0.20
Sodium (mmol/L)	135	135–146
Potassium (mmol/L)	3.9	3.5–4.6
Chloride (mmol/L)	101	96–110
Calcium (mg/dl)	8.9	8.2–10.2
Phosphorus (mg/dl)	3.0	2.5–5.5
Blood urea nitrogen (mg/dl)	38.3	7.0–20.0
Creatinine (mg/dl)	0.85	0.7–1.3
eGFR (ml/minute)	51.9	≥ 60.0
Total protein (g/dl)	6.1	6.5–8.2
Albumin (g/dl)	2.8	3.5–5.5
Total bilirubin (mg/dl)	0.6	0.1–1.2
Aspartate aminotransferase (U/L)	33	10–35
Alanine aminotransferase (U/L)	34	5–40
Alkaline phosphatase (U/L)	245	100–340
Lactate dehydrogenase (U/L)	284	110–220
*y*-Glutamyl transpeptidase (U/L)	12	≤ 60
Creatine kinase (U/L)	132	40–200
Blood count		Reference
Red blood cell (× 10^4^/μL)	341	427–570
Hemoglobin (g/dl)	10.7	13.5–17.6
Hematocrit (%)	31.0	39.8–51.8
MCV (fl)	90.9	82.7–101.6
Platelet (× 10^4^/μL)	23.7	13.1–36.2
White blood cell (μL)	13,380	3900–9800
Neutrophil counts (μL)	11,600	
Diabetes		Reference
Glucose (mg/dl)	142	70–109
Hemoglobin A1c (%)	6.7	4.7–6.2
IRI (μlU/ml)	102.1	3.0–18.0
CPR (ng/ml)	0.63	0.6–1.8
GAD antibody	≤ 5.0	≤ 5.0
IA-2 antibody	≤ 0.4	≤ 0.4
Urinary albumin (mg/gCr)	281.0	
Thyroid		Reference
Free triiodothyronine (pg/ml)	31.7	2.2–4.1
Free thyroxine (ng/dl)	3.43	0.88–1.81
Thyroid-stimulating hormone (μlU/ml)	0.128	0.35–3.73
TSH receptor antibody (IU/L)	0.51	< 2.0
Thyroid stimulating antibody (%)	102	≤ 120
Thyroglobulin (ng/ml)	48,000	≤ 33.70
Thyroglobulin antibody (IU/ml)	457	< 28
Thyroid peroxidase antibody (IU/ml)	8.0	< 16
Endocrine		Reference
Cortisol (μg/dl)	27.5	4.5–21.1
Growth hormone (ng/ml)	0.13	≤ 2.47
Somatomedin C (ng/ml)	39	48–177
Luteinizing hormone (mlU/ml)	18.8	0.8–5.7
Follicle-stimulating hormone (mlU/ml)	5.8	2.0–8.3
Free testosterone (pg/ml)	11.0	4.6–16.9
Prolactin (ng/ml)	9.6	3.6–12.8
Antidiuretic hormone (pg/ml)	2.7	≤ 2.8

*CPR* C-peptide immunoreactivity, *eGFR* estimated glomerular filtration rate, *GAD* glutamic acid decarboxylase, *IA-2* islet antigen 2, *IRI* immunoreactive insulin, *MCV* mean corpuscular volume, *TSH* thyroid-stimulating hormone

According to the diagnostic criteria of the Japan Thyroid Association for thyroid storm [1], he was diagnosed as having thyroid storm 1 (TS1), definite thyroid storm, since he had thyrotoxicosis, a central nervous system symptom (restlessness), fever (38 °C), GI symptoms (nausea, vomiting), and tachycardia (135 beats per minute) in atrial fibrillation. Based on the diagnostic criteria of Burch and Wartofsky for thyroid storm, he scored 60; a score higher than 45 is suggestive of thyroid storm [6]. Graves’ disease was less likely as thyroid-stimulating immunoglobulin was within the normal range, and ^99m^Tc-scintigraphy revealed a quite low uptake. These results indicated that his diagnosis of thyroid storm was due to destructive thyroiditis.

**Fig. 1:**
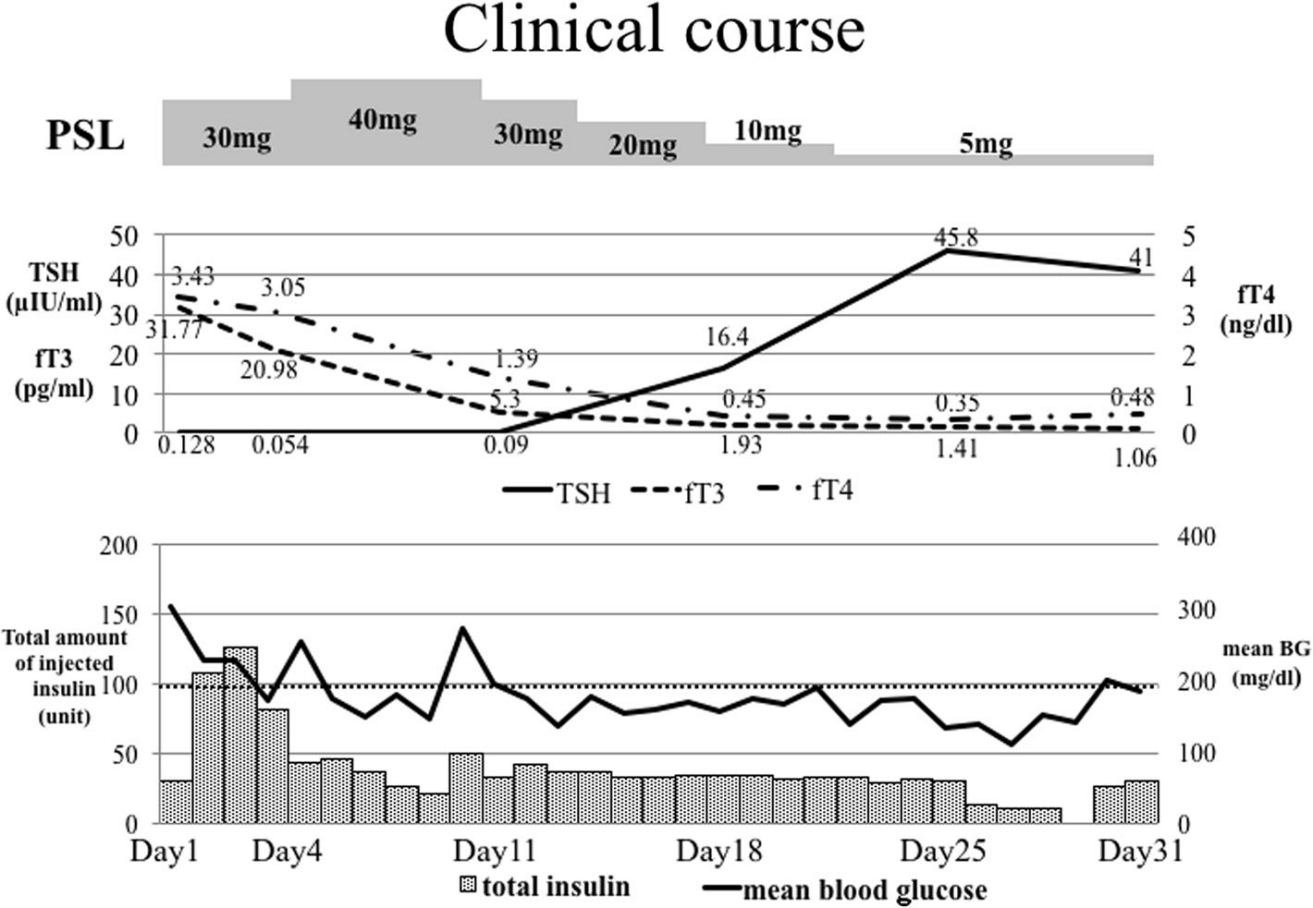
The clinical course of the present case. *BG* blood glucose, *fT3* free triiodothyronine, *fT4* free thyroxine, *PSL* prednisolone, *TSH* thyroid-stimulating hormone

His clinical course is shown in Fig. 1. He was treated with an intravenously administered insulin infusion and intravenously administered fluid therapy. At first, the thyroid storm was treated with orally administered potassium iodide (50 mg every 6 hours) and a short-acting beta-adrenoreceptor blocker, landiolol hydrochloride, was used at 4–10 μg/kg per minute to control his heart rate. The potassium iodide was aborted when he was diagnosed as having distractive thyroiditis. Prednisolone was given at 0.5–0.7 mg/kg per day as a treatment for irAE and thyroid storm. Although previous reports suggested the optimum dosage of prednisolone to be 1–2 mg/kg per day [1, 5], we used a lower dose due to the coexisting and uncontrolled diabetes mellitus. By day 5, his tachycardia had resolved, and the landiolol hydrochloride was discontinued. On day 11, his thyroid function was found to have improved, and the amount of total insulin used to control his blood glucose was decreased. On day 25, he was found to have hypothyroidism, and so we restarted the replacement of thyroid hormone. He was discharged from our hospital on day 35 on daily maintenance insulin injection and levothyroxine sodium hydrate.

## Discussion

Thyroid storm is an endocrine emergency that is characterized by rapid deterioration within days or hours of presentation and is associated with a high mortality [1]. Most cases of thyroid storm are caused by the presence of some triggering condition in conjunction with an underlying thyroid condition. This usually involves untreated or uncontrolled Graves’ disease, but may rarely be due to thyrotoxic disorders such as toxic multinodular goiters, TSH-secreting pituitary adenomas, human chorionic gonadotropin (hCG)-secreting hydatidiform moles, metastatic thyroid cancers, and destructive thyroiditis [1]. Thyroid storm is precipitated by many factors, such as the irregular use or discontinuation of anti-thyroid drugs, infection, diabetic ketoacidosis, surgery, radioiodine therapy, adrenocortical insufficiency, and the administration of iodinated contrast agents. In addition, several drugs that cause thyrotoxicosis as an adverse event, including amiodarone, sorafenib, and ipilimumab, have been reported to precipitate thyroid storm [7, 8]. It is important to point out that our patient was treated with amiodarone for 1 year prior to the initiation of nivolumab and ipilimumab. Amiodarone has been associated with thyroid dysfunction; amiodarone was aborted when the patient was admitted to our hospital. Our patient had thyroid function testing that was within normal range prior to the initiation of nivolumab and ipilimumab; however, we could not have ruled out the possibility that the thyroid storm was precipitated by the treatment of amiodarone. A previous case report of thyroid storm caused by combination therapy with ipilimumab and nivolumab was published [9]. On the other hand, Yu *et al.* also reported a case of thyroid storm in a patient receiving immunotherapy for melanoma with ipilimumab alone [10]. Destructive thyroiditis is more frequent and severe with the combination of ipilimumab and nivolumab compared with ipilimumab monotherapy, but still remains rare (< 1%) with both regimens. Since the evaluation of thyroid function is not routinely performed in most immunotherapy trials, the true incidence is unknown.

Combined immune checkpoint inhibition with ipilimumab and nivolumab produces frequent and durable anti-tumor responses in patients with advanced melanoma and has demonstrated promising activity in other cancers [11]. IrAEs, however, frequently complicate therapy, requiring cessation of therapy in nearly 40% of patients [11, 12]. These events are generally managed with high-dose glucocorticoids although clinically severe, prolonged, and even fatal events rarely occur. Therefore, identifying these severe toxicities is a major priority, even for uncommon events.

Morganstein *et al.* reported the rates of overt thyroid dysfunction in the published phase 3 trials involving ipilimumab and/or nivolumab [13]. Of the 18 patients treated with the combination of ipilimumab and nivolumab, four patients (22.2%) developed hypothyroidism, one of whom had a preceding episode of hyperthyroidism, and two had preceding subclinical hyperthyroidism, none of whom needed anti-thyroid drugs, and two of whom were treated with thyroxine (T4). One patient (5.6%) had subclinical hypothyroidism, and a further four patients (22.2%) had subclinical hyperthyroidism without subsequent hypothyroidism. Overall, 29.5% of patients developed thyroid abnormalities, with the lowest rate of 23% in those treated with ipilimumab and the highest rate of 50% in those treated with combination therapy. Thyroid abnormalities were more common in female patients. Although the majority of abnormalities did not require treatment, this still poses a resource burden in terms of the need for follow-up and repeated measurements of thyroid function. It is notable that the subsets of patients who develop hypothyroidism have a transient initial hyperthyroid phase (often subclinical), highlighting the need for the careful recognition and follow-up of those with thyroid abnormalities. On the other hand, several reports indicated that a few patients with hyperthyroidism due to Graves’ disease, not destructive thyroiditis, have also been rarely reported to have received treatment with ipilimumab [14]. Methimazole appears to be efficacious in the treatment of such patients.

Corticosteroids should be administered as prophylaxis for the relative adrenal insufficiency caused by the hypermetabolic state in thyroid storm. Large doses of corticosteroids have been shown to inhibit both thyroid hormone synthesis and the peripheral conversion of T4 to triiodothyronine (T3) [15]. A high dose of steroid (prednisone at 1–2 mg/kg per day) has been recommended for the treatment of irAE with an immune checkpoint inhibitor [5]. In our case, we reduced the amount of steroid because our patient had uncontrolled diabetes on self-injection of insulin.

Some reports have described that cases of autoimmune diabetes, known as type 1 diabetes, have emerged in association with the use of the anti-PD-1 antibody therapies [16, 17]. Autoimmune diabetes is characterized by the development of an adaptive immune response against specific β-cell antigens. Longitudinal studies in patients have shown that certain autoantibodies, such as anti-IAA, anti-ICA512, and anti-GAD65, define preclinical disease as they are present in the serum for years prior to the onset of symptoms [18]. Half of the previously reported patients who developed insulin-dependent diabetes after anti-PD-1 therapy likewise showed no detectable islet autoantibodies. The pathogenesis in these patients thus seems to differ at least partly from that of conventional autoimmune type 1 diabetes which involves islet autoantibodies. Ansari *et al.* found no correlation between IAA levels and the development of diabetes with blockade of the PD-1–PD-L1 pathway in mice, and some mice developed diabetes despite the apparent absence of autoantibodies [19]. In the present case, all results for islet autoantibodies were negative. However, the present case should not be like a type 1 diabetes mellitus. Although islet autoantibodies remained negative for 3 months after the onset of his symptoms, his insulin secretion improved back to previous levels before the initiation of treatment with checkpoint inhibitors. In this case, thyrotoxicosis induced by checkpoint inhibitors worsened his blood glucose control so that he needed more insulin injections.

## Conclusions

Immune checkpoint inhibitors will be widely used for melanoma. It might be important to be aware of the possibility of thyroid storm induced by combination therapy of ipilimumab and nivolumab.
